# Short-term changes of choroidal vascular structures after phacoemulsification surgery

**DOI:** 10.1186/s12886-018-0749-7

**Published:** 2018-03-23

**Authors:** Haisong Chen, Zheming Wu, Yun Chen, Manshan He, Jiawei Wang

**Affiliations:** 1 Guangzhou Aier Eye Hospital, Guangzhou, China; 2 Eye Center of Shandong University, The Second Hospital of Shandong University, Shandong University, Jinan, 250000 China

**Keywords:** Choroid, Vascular structures, Phacoemulsification

## Abstract

**Background:**

To evaluate the changes of choroidal vascular structures in patients after phacoemulsification surgery.

**Methods:**

A self-control study was conducted on 36 eyes of 36 patients who had uneventful phacoemulsification. Choroidal images were acquired preoperatively, 7 days (D7), 1 month (M1), and 3 months (M3) after surgery from enhanced depth imaging (EDI) optical coherence tomography (OCT) scans. Choroidal vascularity index (CVI) was used to assess vascular status of the choroid using image binarization by the Niblack method. The postoperative values of mean CVI were compared with baseline by paired t-test. Univariate and multiple linear regression analyses were performed to determine the associations between CVI and other factors.

**Results:**

The mean age of the recruited patients was 63.1 ± 6.9 years. The mean CVI at baseline was 60.1 ± 5.5%. After surgery, the CVI significantly increased to 61.7 ± 5.3% at D7, 63.6 ± 4.4% at M1 and 64.8 ± 4.0% at M3 (*p* = 0.035, 0.0006, < 0.0001, respectively). Univariate and multiple regression analysis revealed a positive association between CVI and subfoveal choroidal thickness (SFCT) at pre-operation and no significant association with age, axial length (AL), intraocular pressure (IOP) and gender at all timepoints.

**Conclusions:**

Phacoemulsification induced increased CVI in patients diagnosed with cataract. Evaluation of the long-term change of CVI following surgery may provide valuable information for studying the relationship between phacoemulsification and disorders of the choroid.

## Background

Cataract with phacoemulsification surgery is the most extensively performed eye surgery. There are more than 1300 cases per million people undergoing phacoemulsification surgery per year in China and greater than 5000 cases per million people per year in Europe, America and India [1]. Phacoemulsification surgery is safe and generally associated with successful visual outcomes.

Choroid, the highest blood circulation in the human body, is composed of blood vessels, connective tissues, nerves, melanocytes and extracellular fluid. A great deal of analysis and research indicates that even uncomplicated phacoemulsification induces disorders of the choroid, especially an increase in the choroid thickness [2–4]. However, only the choroidal thickness does not supply convincing evidence on what structures change, especially about the blood volume within the choroid in patients after Phacoemulsification surgery.

Further morphological and vascular analyses of the choroid may certify the change of choroidal blood volume in patients after Phacoemulsification surgery. With the advent of enhanced depth imaging (EDI) optical coherence tomography (OCT), it is possible to assess the choroidal stromal and vascular structures. Recently, application of image binarization of choroid structures has further provided a novel measure index for vascular status of the choroid [5]. To the best of our knowledge, there is no report about the change of choroidal vascular structures after Phacoemulsification surgery.

In the current study, we aimed to determine the influence of phacoemulsification on the proportion of choroidal vascular structures in patients after surgery. The choroidal vascularity index (CVI) from EDI-OCT scans will be used and we speculated that CVI may provide more additional information about the morphology and physiology of the choroid and may be useful to interpret the disorders of the choroid after cataract surgery.

## Methods

Thirty-six healthy patients undergoing uncomplicated Phacoemulsification surgery were recruited for this self-controlled case series study. All the patients were recruited consecutively (from October 2016 to December 2016) from the cataract department of Guangzhou Aier Eye Hospital and signed the consent form after a fullest explanation of the purpose and procedures of the study. The study was adhered to the provisions of the Declaration of Helsinki for research involving human subjects and was approved by the Ethical Review Committee of Guangzhou Aier Eye Hospital.

All the study participants were healthy individuals with no history of ocular disease or visual symptoms; aged at least 40 years; intraocular pressure (IOP) < 21 mmHg; normal appearance of optic nerve head; normal anterior chamber angles; Patients were excluded if they had glaucoma, high myopia or hyperopia (magnitude exceeding ±6 diopters (D) of spherical equivalent refraction), AMD, or other retinal diseases that could interfere the choroidal thickness. The diagnosis of glaucoma was based on the findings from gonioscopy, optic disc characteristics, and visual fields results. Patients with severe systemic diseases, such as diabetes mellitus, rheumatism, or malignant tumors, serious opacity of refractive media or unstable fixation that could prevent EDI-OCT measurement were also excluded.

All the patients underwent a comprehensive ophthalmologic examination, including IOP measurement using Goldmann applanation tonometry, autorefraction examination, measurement of visual acuity and a best-corrected visual acuity (BCVA), axial length using ocular biometry (IOL Master, Zeiss, Germany), fundus examination and EDI-OCT measurement (Spectralis, Heidelberg Engineering, Heidelberg, Germany) before surgery and postoperatively at 7 days (D7), month 1 (M1) and months 3 (M3).

All patients received standard phacoemulsification surgery through clear corneal incisions under superficial anesthesia (0.5% Proparacaine hydrochloride Eye Drops, Alcon, Fort Worth, TX). All phacoemulsification surgeries was performed by the same experienced surgeon (HSC) using the Infiniti system® (Alcon Labs Inc). In all cases, after removal of the lens cortex, a foldable intraocular lens was implanted uneventfully in the capsular bag. Within 1 month after surgery, Tobradex (0.3% tobramycin and 1% dexamethasone, Alcon, Fort Worth, TX) eye drops were applied four times a day, a non-steroidal anti-inflammatory eye drops (Pranoprofen Eye Drops, Senju Pharmaceutical Co.,Ltd. Osaka, Japan) were applied four times a day, and TobraDex eye ointment (Alcon, Fort Worth, TX) was applied once every evening before bed.

### Image acquisition

EDI-OCT scans of the macular were performed for the operated eye using the EDI mode of SD-OCT (Spectralis, Heidelberg Engineering, Heidelberg, Germany). Horizontal 6-mm line scans centred on the fovea were acquired. Due to the diurnal variation of choroidal thickness, all the measurements were performed at the same time of the day (08:00 AM~ 12:00 AM) and accomplished in triplicate by two independent examiners. The sections going directly through the center of the fovea were selected for further analysis. The subfoveal choroidal thickness (SFCT) was measured using the in-built calipers tool. SFCT was defined as the vertical distance between the outer surface of the retinal pigment epithelium and the choroidal–sclera interface [6].

### Procedures of image binarization

**Fig. 1:**
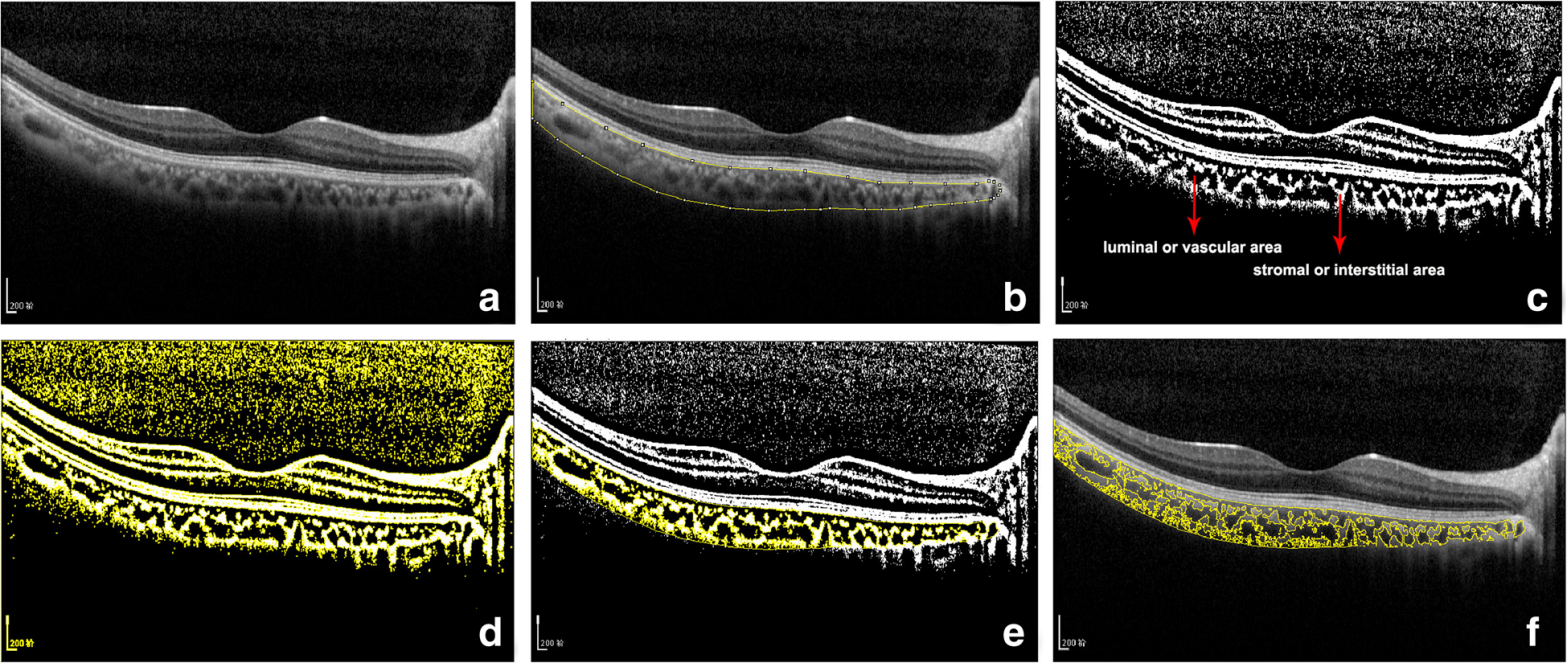
Image binarization for choroid with Niblack auto local thresholding technique. **a** Original EDI-OCT scan image. **b** Manual segmentation of the choroidal area with one central scan passing through the fovea. **c** Conversion of the image with the Auto Local Threshold tool. **d** Clear segmentation of black and white areas on the choroid with Niblack autolocal threshold. **e** binarized image was reconverted back to RGB image. **f** binarized image over the original EDI-OCT scan

Image binarization of the subfoveal choroidal area was performed by one public domain software, Image J (version 1.47, provided in the public domain by the National Institutes of Health, Bethesda, MD, USA; http://imagej.nih.gov/ij/) [7, 8]. In brief, the images with one central scan passing through the fovea were chosen. The region of interest was manually selected using the polygon tool and added to ROI manager. After measuring the brightness of the selected luminal areas of the original OCT, the average brightness was set at the minimum value to minimize the noise in the OCT image. Then the original images were converted to 8 bits and adjusted by the Niblack Auto Local Threshold. The binarized image was converted to RGB (red, green, blue) image again, and the luminal area was determined using the Threshold Tool. After the image binarization, the total circumscribed area (TCA) and area of dark pixels were calculated. The dark pixels represent the luminal or vascular area (LA) and stromal or interstitial area (SA) was defined as the area of light pixels (Fig. 1). CVI was defined as the proportion of LA to TCA.

### Inter-rater and intra-rater agreement

All the preoperative images were initially segmented by two graders to evaluate the inter-rater agreement (HSC and JWW). The same amount of images was segmented by one grader (JWW) after an interval of one week to determine intra-rater reliability. Absolute agreement model of the intra-class correlation coefficient (ICC) was used for the intra- and inter-rater reliability for the image binarization. ICC value of 0.81–1.00 indicates good agreement. The mean difference between the measurements was calculated by Bland-Altman plot analysis, which was constructed using MedCalc version 17.5.3 (Medcalc Statistical Software, Ostend, Belgium) software. After obtaining good inter-rater and intra-rater agreement, all the image binarization was performed by single author (JWW).

### Statistical analysis

All statistical analyses were performed using SPSS software version 20.0 (IBM-SPSS, Chicago, Illinois, USA). Normally distributed data were expressed as mean ± standard deviation (SD). Each postoperative value was compared with baseline by paired t-test. Univariate and multiple linear regression analyses were performed to determine the associations between CVI and other factors. Values of *p* < 0.05 were considered to be statistically significant.

## Results

A total of 36 patients (20 male and 16 female) were finally recruited for the current study and the demographic characteristics of the patients are shown in Table 1. Mean age of the volunteers was 63.1 ± 6.9 years (range, 49~ 78).

**Table 1:** Demographics characteristics of the recruited subjects

Characteristics	Mean ± SD			
	Baseline	D7	W1	W3
Axial length, mm	23.55 ± 1.58	23.37 ± 1.65	23.30 ± 1.58	23.11 ± 1.56
Intraocular pressure, mmHg	14.19 ± 2.13	13.18 ± 1.91	12.54 ± 1.62	11.93 ± 1.80
Subfoveal choroidal thickness (SFCT),um	234.8 ± 42.49	239.7 ± 40.61	266.6 ± 37.71	276.3 ± 36.20
Age, yrs	63.1 ± 6.9			
Gender, male (%)	20 (55.6%)			

Data were expressed as mean ± standard deviation (SD)

**Fig. 2:**
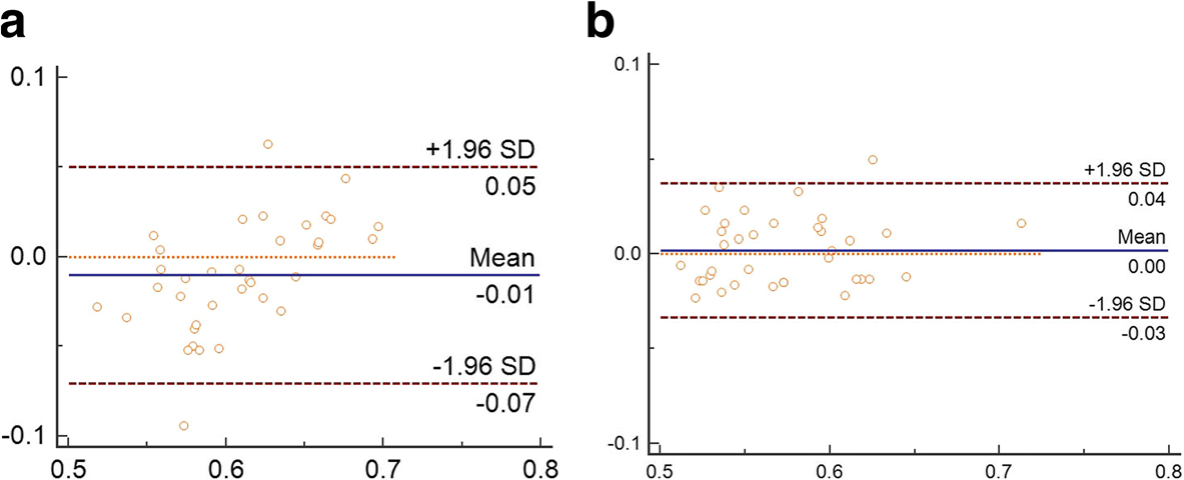
Bland-Altman plot analysis of the intra- and inter-rater agreement. **a** and **b** shows the high reliability of the inter-rater and intra-rater agreement for the image binarization and CVI calculation

The inter-rater agreement for CVI was 0.932 (95% CI: 0.866–0.965) and the inter-rater reliability was 0.959 (95% CI: 0.920–0.979), which indicates excellentagreement for image binarization and CVI calculation. Bland Altman plot analysis was constructed to display the high agreement (Fig. 2).

**Fig. 3:**
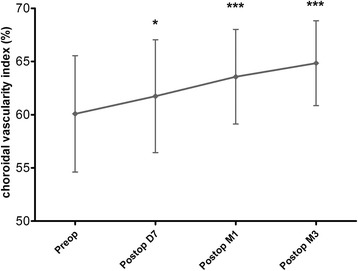
Time course of CVI before and after phacoemulsification surgery. CVI = Choroidal vascularity index

The baseline CVI in patients was 60.1 ± 5.5%. After surgery, the CVI significantly increased to 61.7 ± 5.3% at D7, 63.6 ± 4.4% at M1 and 64.8 ± 4.0% at M3 (*p* = 0.035, 0.0006, < 0.0001 for D7, M1 and M3 when compared with the preoperative values). The greatest progression of CVI was observed between D7 and M1 after surgery (Fig. 3).

Univariate linear regression analysis revealed a positive association between CVI and SFCT at baseline and M1 postoperative follow-up. Age was found to be related with CVI at W1 after surgery. However, in the multiple regression model, only SFCT was significantly associated with CVI. Univariate and multivariate linear regression analyses revealed no significant association of CVI with AL, IOP and gender (Table 2).

**Table 2:** Univariate and multivariate linear regression analyses of age, gender and ocular factors associated with choroidal vascularity index

		Univariate			Multivariate		
		Unstandardized β	Standardized β	*P*-value	Unstandardized β	Standardized β	*P*-value
Age	Pre	0.053	0.067	0.698	0.092	0.115	0.462
	D7	0.032	0.041	0.810	0.048	0.062	0.721
	W1	0.225	0.346	0.039	0.163	0.251	0.119
	W3	0.015	0.026	0.880	0.024	0.041	0.818
Gender	Pre	3.520	0.324	0.054	3.307	0.305	0.057
	D7	3.056	0.291	0.085	2.246	0.214	0.234
	W1	0.349	0.040	0.819	−0.177	−0.020	0.898
	W3	0.237	0.030	0.862	0.487	0.062	0.725
Axial length, mm	Pre	−0.171	− 0.288	0.775	− 0.524	− 0.204	0.202
	D7	0.550	0.171	0.317	0.660	0.206	0.271
	W1	0.424	0.151	0.380	0.579	0.206	0.231
	W3	−0.037	−0.014	0.933	− 0.175	− 0.069	0.706
IOP, mmHg	Pre	−0.575	−0.233	0.190	−0.524	− 0.204	0.202
	D7	0.145	0.052	0.763	0.152	0.055	0.756
	W1	−0.731	−0.266	0.116	−0.518	−0.189	0.229
	W3	−0.702	−0.317	0.060	−0.743	− 0.335	0.065
SFCT, μm	Pre	0.051	0.399	0.016	0.053	0.413	0.026*
	D7	0.029	0.223	0.190	0.033	0.253	0.168
	W1	0.044	0.372	0.025	0.048	0.406	0.02*
	W3	0.009	0.086	0.618	0.010	0.092	0.603

*Adjusted for variables with a *p*-value< 0.05 in the univariate analysis. β, regression coefcient

## Discussion

**Fig. 4:**
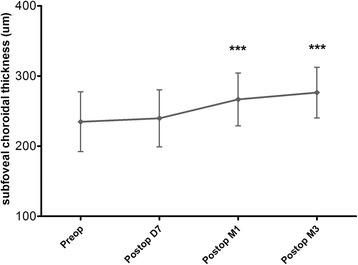
Time course of SFCT before and after phacoemulsification surgery. SFCT = subfoveal choroidal thickness

A number of publications have reported the possible influence of cataract surgery on the choroid [2, 3, 9, 10]. Aslan BS et al. [9] have investigated the effects of uneventful phacoemulsification surgery on choroidal thickness using spectral domain optical coherence tomography (SD-OCT). They found that phacoemulsification may cause significant increase in choroid at all regions evaluated at 1 month postoperative follow-up. Yılmaz T et al. [10] have reported a long-term change in SFCT after cataract surgery. They have measured the SCT at baseline and postoperatively at week 1 and months 1, 3, 6 and 12 and the results indicated that uncomplicated phacoemulsification induced insignificant increases in SFCT and this did not return to baseline during follow-up. In our study, we found that the mean SFCT, as tested by EDI-OCT, significantly increased after surgery, which was consistent with the published literatures (Fig. 4). As well known, the choroid is predominantly composed of blood vessels surrounded by stromal tissue. Although many studies, including our study, have described the increased SCT after phacoemulsification, nobody really knows which structures within the choroid increased. To answer this question, we used the binarization of EDI-OCT images and firstly attempted to assess the changes of choroidal vascular and stromal structures following cataract surgery.

With the advent of EDI-OCT, it is possible to analyze the structural changes allowing for quantitative measurements of choroidal vasculature in patients. Recently, an OCT based metric termed CVI has been used to assess the choroidal vascularity, using the image binarization technique for EDI-OCT scans [5]. CVI is more stable and less interferences from physiologic factors as opposed to the thickness of choroid [5, 7, 11]. In our previous study, we have found that SFCT is affected by many physiological factors, like AL and gender [12]. When comparing the factors influencing CVI, we found no significant association of CVI with AL, IOP and gender and there were positive associations between CVI and SFCT at pre-operation and 1 month postoperative follow-up. CVI was affected by few variables and demonstrated greater stability than SFCT. Measuring of the CVI would provide deeper understanding of the vascular structural changes in the process of choroid diseases, and therefore may be more informative compared to SFCT measurements alone.

By calculating the CVI, it is able to determine if there was an increase or decrease in vascularity and provide us more information on the proportion of vascularity in the choroid. Agrawal R and collaborators [13] found that eyes with acute central serous chorioretinopathy (CSC) had significantly higher CVI compared with their fellow eyes and age-matched healthy subjects. They demonstrated that CVI might be useful for the early diagnosis of CSC and to be a therapeutic index for the treatment response after laser photocoagulation or photodynamic therapy. In patients diagnosed with exudative age-related macular degeneration (AMD), there was a significantly lower CVI and CVI was probably a potential noninvasive tool for studying structural changes in choroid and exudative AMD development monitored [14]. Therefore, CVI is considered to be a relatively stable index to monitor the progression of choroidal diseases [15–17].

Phacoemulsification is the most frequently performed surgical intervention worldwide and considered to be safe and effective. However, in the past, concern has been raised about the association between cataract surgery with the incidence or progression of AMD. Disorders of the choroid after Phacoemulsification may cause the onset of many choroid diseases, including AMD. Some researchers have raised concerns that phacoemulsification may constitute a risk factor for the development of exudative AMD [18–20]. In contrast, recent evidence does not find the surgery to cause or worsen the progression of AMD [21–24]. In our study, the CVI was 60.1 ± 5.5% in the baseline and significantly increased D7, M1 and M3 after the surgery. The greatest progression of CVI was observed between D7 and M1 after surgery. Therefore, our data suggested that phacoemulsification seemed to be able to induce the expansion of choroidal vascular structures within 3 months after surgery. As a more stable parameter for disease monitoring, it will be very useful to evaluate CVI of patients after phacoemulsification at further follow-up over a longer period. Evaluation of CVI may provide further evidence about the relationship between phacoemulsification and AMD.

We found that phacoemulsification induced progressive increases in CVI. On the other hand, the surgery increased the proportion of vascular structures in the choroid. We suspect that the increased CVI may depend crucially on the choroidal inflammation induced by surgical trauma [25]. With the disruption to the blood-aqueous barrier, the inflammatory mediators in the aqueous humor pass through the vitreous to the retina and choroid, subsequently leading to the change of choroidal vascular structures. Another possibility is the IOP decrease after cataract surgery. The increased ocular perfusion pressure caused by reduced IOP may induce the increased CVI in the early period after phacoemulsification. However, the concrete mechanism has yet to be fully explained and further study is needed.

There are some limitations in the current study. Firstly, although this binarization technique is valid and widely used, there is no concrete evidence that the dark areas represented the vascular areas. Secondly, the postoperative anti-inflammatory eye drops may affect the CVI evaluation; Thirdly, the average ultrasonic emulsification time (UST) and the cumulative dissipated energy (CDE) were not recorded, which may also influence the CVI analysis. Lastly, the cohort of patients was not large enough and we only assessed the short-term changes of CVI after surgery. The future bigger sample research and longer follow-up periods are needed, especially to clarify the relationship between phacoemulsification and the frequency of disorders of the choroid such as AMD.

## Conclusions

In the follow-up study, we firstly used CVI to assess the change of choroidal vascular structures in patients undergoing phacoemulsification surgery. Our results showed that the proportion of vascularity in the choroid, termed as CVI, significantly increased within 3 months following surgery. Evaluation of CVI may provide valuable information for studying the relationship between phacoemulsification and disorders of the choroid such as AMD.

## Abbreviations

AL: Axial length
AMD: Age-related macular degeneration
CSC: Central serous chorioretinopathy
CVI: Choroidal vascularity index
EDI: Enhanced depth imaging
IOP: Intraocular pressure
LA: Luminal or vascular area
OCT: Optical coherence tomography
SA: Stromal or interstitial area
SFCT: Subfoveal choroidal thickness
TCA: Total circumscribed area
